# Efficacy of Astaxanthin for the Treatment of Atopic Dermatitis in a Murine Model

**DOI:** 10.1371/journal.pone.0152288

**Published:** 2016-03-29

**Authors:** Yoko Yoshihisa, Tsugunobu Andoh, Kenji Matsunaga, Mati Ur Rehman, Takashi Maoka, Tadamichi Shimizu

**Affiliations:** 1 Department of Dermatology, Graduate School of Medicine and Pharmaceutical Sciences, University of Toyama, Sugitani, Toyama, Japan; 2 Department of Applied Pharmacology, Graduate School of Medicine and Pharmaceutical Sciences, University of Toyama, Sugitani, Toyama, Japan; 3 Department of Radiological Sciences, Graduate School of Medicine and Pharmaceutical Sciences, University of Toyama, Sugitani, Toyama, Japan; 4 Division of Food Function and Chemistry, Research Institute for Production Development, Shimogamo-morimoto-cho, Sakyo-ku, Kyoto, Japan; INSERM-Université Paris-Sud, FRANCE

## Abstract

Atopic dermatitis (AD) is a common chronic inflammatory skin disease associated with various factors, including immunological abnormalities and exposure to allergens. Astaxanthin (AST) is a xanthophyll carotenoid that has recently been demonstrated to have anti-inflammatory effects and to regulate the expression of inflammatory cytokines. Thus, we investigated whether AST could improve the dermatitis and pruritus in a murine model of AD using NC/Nga mice. In addition to a behavioral evaluation, the effects of AST on the AD were determined by the clinical skin severity score, serum IgE level, histological analyses of skin, and by reverse transcription-PCR and Western blotting analyses for the expression of inflammation-related factors. AST (100 mg/kg) or vehicle (olive oil) was orally administered once day and three times a week for 26 days. When compared with vehicle-treated group, the administration of AST significantly reduced the clinical skin severity score. In addition, the spontaneous scratching in AD model mice was reduced by AST administration. Moreover, the serum IgE level was markedly decreased by the oral administration of AST compared to that in vehicle-treated mice. The number of eosinophils, total and degranulated mast cells all significantly decreased in the skin of AST-treated mice compared with vehicle-treated mice. The mRNA and protein levels of eotaxin, MIF, IL-4, IL-5 and L-histidine decarboxylase were significantly decreased in the skin of AST-treated mice compared with vehicle-treated mice. These results suggest that AST improves the dermatitis and pruritus in AD via the regulation of the inflammatory effects and the expression of inflammatory cytokines.

## Introduction

The skin is exposed to endogenous and environmental pro-oxidant agents, as a result, they cause the upregulation of reactive oxygen species (ROS). The resulting oxidative stress damages proteins, lipids and DNA. An imbalance between ROS and antioxidants can lead to an elevated oxidative stress level [1].

Atopic dermatitis (AD) is a chronic inflammatory skin disease associated with various factors, including immunological abnormalities and exposure to allergens that contribute to the pathogenesis and development of skin lesions. Some evidence indicates that oxidative stress is involved in the cutaneous damage in AD. Enhancement and attenuation of the antioxidant defenses have been shown to be associated with the amelioration and exacerbation of AD, respectively [2]. AD patients were more prone to damage caused by ROS or oxidants than controls. It was also reported that oxidative stress was involved in the pathophysiology of chronic AD without recent flare-ups [3]. Tsuboi *et al*. reported that adult patients with AD excreted significantly higher levels of 8-hydroxy-2'-deoxyguanosine (8-OHdG), an established marker of oxidative stress, in the urine compared with their corresponding controls [4]. High urinary levels of 8-OHdG in children with chronic AD were also reported [2]. The mean concentration of 8-OHdG was 1.6-fold higher in AD patients compared with healthy controls, and the use of anti-oxidants or nitric oxide (NO) pathway modulators have been considered as a potential therapeutic strategy [5]. Taken together, these findings indicate that enhanced oxidative stress can be particularly harmful for AD patients.

Astaxanthin (AST), 3,3’-dihydroxy-β-carotene-4,4’-dione, a carotenoid without vitamin A activity [6], has potential clinical applications due to its antioxidant activity, which is higher than that of β-carotene and α-tocopherol [6–8]. In addition, it has many highly potent pharmacological effects, including anti-tumor, anti-cancer, anti-diabetic and anti-inflammatory activities [8–11]. AST has also been reported to inhibit the production of ROS, and protects bovine oviduct epithelial cells from NO-induced oxidative stress [12]. In addition, AST inhibits the production of inflammatory mediators by inhibiting the NF-kB signaling pathway [13, 14]. The topical administration of AST prevents ultraviolet (UV)-induced photokeratitis in mice by decreasing the oxidative stress in the irradiated corneas [15]. We also recently demonstrated that AST treatment decreases the iNOS expression and inhibits UV-induced apotosis in keratinocytes [16].

The present study was designed to investigate whether AST could be able to suppress the pruritus and decrease the skin severity of AD like lesions in a murine model of AD using NC/Nga mice.

## Materials and Methods

### Reagents

AST was provided by Fuji Chemical Industry Co., Ltd. (Toyama, Japan). The Isogen RNA extraction kit was obtained from Nippon Gene (Tokyo, Japan). M-MLV reverse transcriptase was purchased from GIBCO (Grand Island, NY). Ex-Taq DNATM polymerase was purchased from TaKaRa Bio (Shiga, Japan). The anti-MIF polyclonal antibody (pAb) was obtained from R&D Systems, Inc. (Minneapolis, MN). The anti-eotaxin pAb and anti-IL-4 pAb were obtained from Abcam Ltd. (Cambridge, UK). The anti-L-histidine decarboxylase (HDC) pAb was obtained from Progen Biotechnik (Heidelberg, Germany). The anti-IL-5 pAb, anti-IFN-γ pAb and anti-β-actin pAb were purchased from Santa Cruz Biotechnology, Inc. (CA). The Western blot detection system was obtained from Cell Signaling Technology (Beverly, MA). The IgE ELISA kit was obtained from DS Pharma Biomedical Co., Ltd. (Osaka, Japan). All other reagents were of analytical grade.

### Mice

Male NC/Nga mice (eight weeks old at the start of the experiments: Japan SLC, Shizuoka, Japan) were used for the study. The induction of AD using mites was performed as described previously [17]. Mice were kept under controlled temperature (21–23°C) and humidity (45–65%) conditions. The room was lit from 07:00 to 19:00 hours. Food and water were provided ad libitum.

### Ethics Statement

This study was carried out in strict accordance with the recommendations in the Institute for animal experiments at University of Toyama. The protocol was approved by the committee on the ethics of animal experiments of University of Toyama.

### Administration of AST

AST (100 mg/kg) or vehicle (olive oil) was orally administered three times a week for 26 days by gavage (oral zoned needle). Each group consisted of five mice.

### Evaluation of the clinical skin severity score

Mice were observed and the skin conditions were scored. The total clinical severity for AD-like lesions was defined as the sum of the individual scores graded as 0 (none), 1 (mild), 2 (moderate) and 3 (severe) for each of five signs and symptoms (itching, erythema, edema, excoriation/erosion and scaling/dryness) [17].

### Behavioral evaluation

Scratching behavior was observed as described previously [18]. Briefly, the animals were placed individually in acrylic cages composed of four equal-sized cells (13×9×40 cm) for at least 1 hour for acclimation. Then, their behaviors were videotaped for 1 hour with personnel kept out of the observation room. Spontaneous scratching toward any rostral regions of the body by the hind paws was counted. As mice make several rapid scratching movements for periods of about 1 sec, a series of these movements was counted to be one bout of scratching [19].

### Determination of the IgE level

Blood (20 μl) was collected from the hearts using a capillary glass tube under ether anesthesia, and was centrifuged at 4°C at 5,000 rpm for 20 min. The serum was kept at -80°C until it was assayed. The total IgE in the serum was determined with an ELISA kit.

### Plasma sample preparation and high-pressure liquid chromatography (HPLC) analysis

The AST content in the plasma and skin tissues was quantified using high performance liquid chromatography (HPLC). Blood samples were treated with EDTA and citrate-theophylline-adenosine-dipyridamole (BD Biosciences, Tokyo, Japan) and centrifuged at 2,500×g for 30 minutes to obtain plasma and then stored at −80°C until HPLC measurement. Plasma treated with 5 mL of hexane/ether (8:2, v/v) was subjected to preparative HPLC, while the skin tissues were evaporated until dry and subsequently expressed with 3 mL of acetone and homogenized. The residue was filtered, and the solution was subjected to HPLC on a Shimadzu SPD-6AV spectrophotometer (Shimadzu, Kyoto, Japan) set at 470 nm. The column used was a Cosmosil 5SL-II column (4.6×250 nm, Nacalai Tesque, Kyoto, Japan) with a mobile phase of acetone/hexane (2:8). A low rate was employed (1.0 ml/min). The AST content was quantified relative to calibration with that observed in a standard sample.

### Histological analysis

Samples of the back skin of the mice (at 0 or 26 days) were fixed with 10% formalin in neutral buffered solution, torn into strips and embedded in paraffin. The sections of 2 μm thickness were stained with hematoxylin and eosin for eosinophils, or toluidine blue for mast cells. Sections were then observed to detect non-degranulated (S1A Fig) and degranulated (S1B Fig) status of mast cells. Cells were counted at a magnification of ×400 and were expressed as the total number of cells and degranulated mast cells in five fields in each mouse (five sections per mouse, five mice per group).

### Reverse transcription-PCR analysis

Total RNA was extracted from each mouse skin sample. RNA reverse transcription was performed with M-MLV reverse transcriptase using random hexamer primers and subsequent amplification using Taq DNA polymerase. PCR was carried out for 35 cycles with denaturation at 98°C for 10 sec, annealing from 55°C for 30 sec and extension at 72°C for 1 min using a thermal cycler (PE Applied Biosystems Gene Amp PCR system 9700). The mouse MIF primers used in the present study were 5’-GTTTCTGTCGGAGCTCAC-3’ (55–72) (forward) and 5’-AGCGAAGGTGGAACC GTTCCA-3’ (215–236) (reverse). The eotaxin primers used were: 5’-CCAAGGACTTGGCTTCATGTAG-3’ (438–459) (forward) and 5’-ATTCTGGCTTGGCATGGTAGC-3’ (912–932) (reverse). The IL-4 primers used were: 5’-ACGGCACAGAGCTATTGATG-3’ (71–90) (forward) and 5’-ATGGTGGCTCAGTACTACGA-3’ (505–524) (reverse). The IL-5 primers used were: 5’-AGGATGCTTCTGCACTTGA-3’ (50–68) (forward), 5’-ACACCAAGGAACTCTTGCA-3’ (396–414) (reverse). The HDC primers used were: 5’-AGCACAAGCTGTCGTCCTTT-3’ (320–339) (forward) and 5’-GTGGATCACGAAGACCCTGT-3’ (514–533) (reverse). The IFN-γ primers used were: 5’-GCTCTG AGACAATGAACGCT-3’ (98–117) (forward) and 5’-AAAGAGATAATCTGGCTCTG C-3’ (306–326) (reverse). GAPDH was used as a positive control. The primers used to amplify GAPDH were: 5’-GAAGGTCGGTGTGAACGGATTTG-3’ (6–28) (forward) and 5’-GTCCACCACCCTGTTGCTGTAGC-3’ (949–971) (reverse). After PCR, the amplified products were analyzed by 2% agarose gel electrophoresis.

### Western blot analysis

The skin samples were lysed in RIPA buffer (1M Tris-HCA, 5M NaCl, 1% Nonidet P-40 (v/v), 1% sodium deoxycholate, 0.05% SDS, 1mM phenylmethyl sulfonyl fluoride) for 20 min. After brief sonication, the lysates were centrifuged at 12,000 rpm for 10 min at 4°C, and the protein content in the supernatants was measured using a Bio-Rad protein assay kit (Bio-Rad, Hercules, CA). The protein lysates were denatured at 96°C for 5 min after mixing with 5 μL of sodium dodecylsulfate (SDS) loading buffer, were applied on an SDS polyacrylamide gel for electrophoresis and were transferred to nitrocellulose membranes. A Western blot analysis was carried out to detect the expression levels of eotaxin, MIF, IL-4, IL-5, IFN-γ and HDC using specific antibodies. Band signals were visualized on X-ray film using chemiluminescence ECL detection reagents (Amersham Biosciences, Buckinghamshire, UK). The relative amounts of proteins associated with specific antibodies were normalized according to the intensities of β-actin. The band density was quantified by a BIO-ID image analyzer, and the relative amounts of proteins associated with specific antibodies were normalized according to the intensities of β-actin.

### Statistical analysis

The values are expressed as the means ± SD of the respective test or control group. The statistical significance of differences between the control and test groups was evaluated by either Student’s t-test, a two-way repeated measures ANOVA or Dunnett’s multiple comparison.

## Results

### AST treatment improved the AD symptoms in the NC/Nga mice

**Fig 1:**
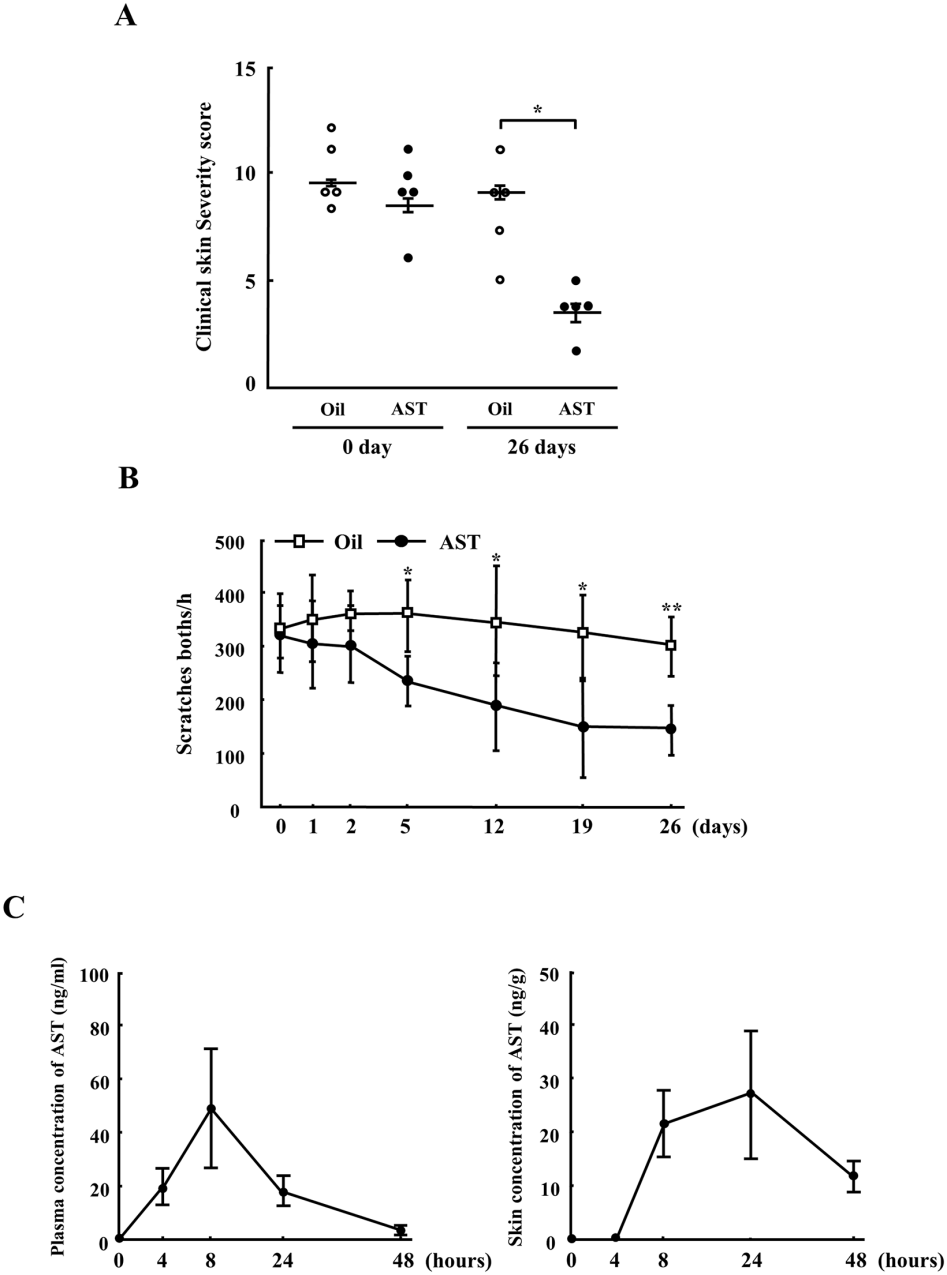
Effects of AST on the clinical severity and scratching behavior of dermatitis and the concentrations of AST in the NC/Nga mice. The clinical skin severity scores (A) and scratching behavior (B) of the NC/Nga mice after the oral administration of oil or AST (100 mg/kg) for 26 days. (C) The concentrations of AST in the NC/Nga mice after the oral administration of AST (100 mg/kg) for 48 hours. Changes in the concentrations of AST in the plasma and skin of the NC/Nga mice. The results are given as the mean ± SD for five mice in each group.*p<0.01.

We first utilized the clinical severity scores to compare the AD-like skin lesions in the NC/Nga mice. On day 0, the skin severity scores of both groups of mice were similar. The skin severity scores of the AST-treated mice were significantly decreased at 26 days compared to the vehicle-treated mice (Vehicle: 9.0 ± 0.6, AST: 3.8 ± 0.7, *p<0.01) (Fig 1A).

### AST treatment suppressed the scratching behavior and changed the plasma and skin concentrations of AST in the NC/Nga mice

We next investigated the effects of AST on the scratching behavior in NC mice. Fig 1B shows the number of spontaneous scratching bouts on the rostral part of the body with the hind paws in individual mice. A decrease in scratching behavior was observed after AST treatment, starting on day five and lasting until day 26, compared to that noted for vehicle treatment (*p<0.05, **p<0.01).

The concentration of AST in the plasma reached 49.0 ± 21.4 eight hours after the oral administration of AST and then decreased until 48 hours in a time-dependent manner. On the other hand, the concentrations of AST in the skin tissues remained elevated until 24 hours after the oral administration of AST (Fig 1C).

### The effects of AST on the histological features of AD in the NC/Nga mice

**Fig 2:**
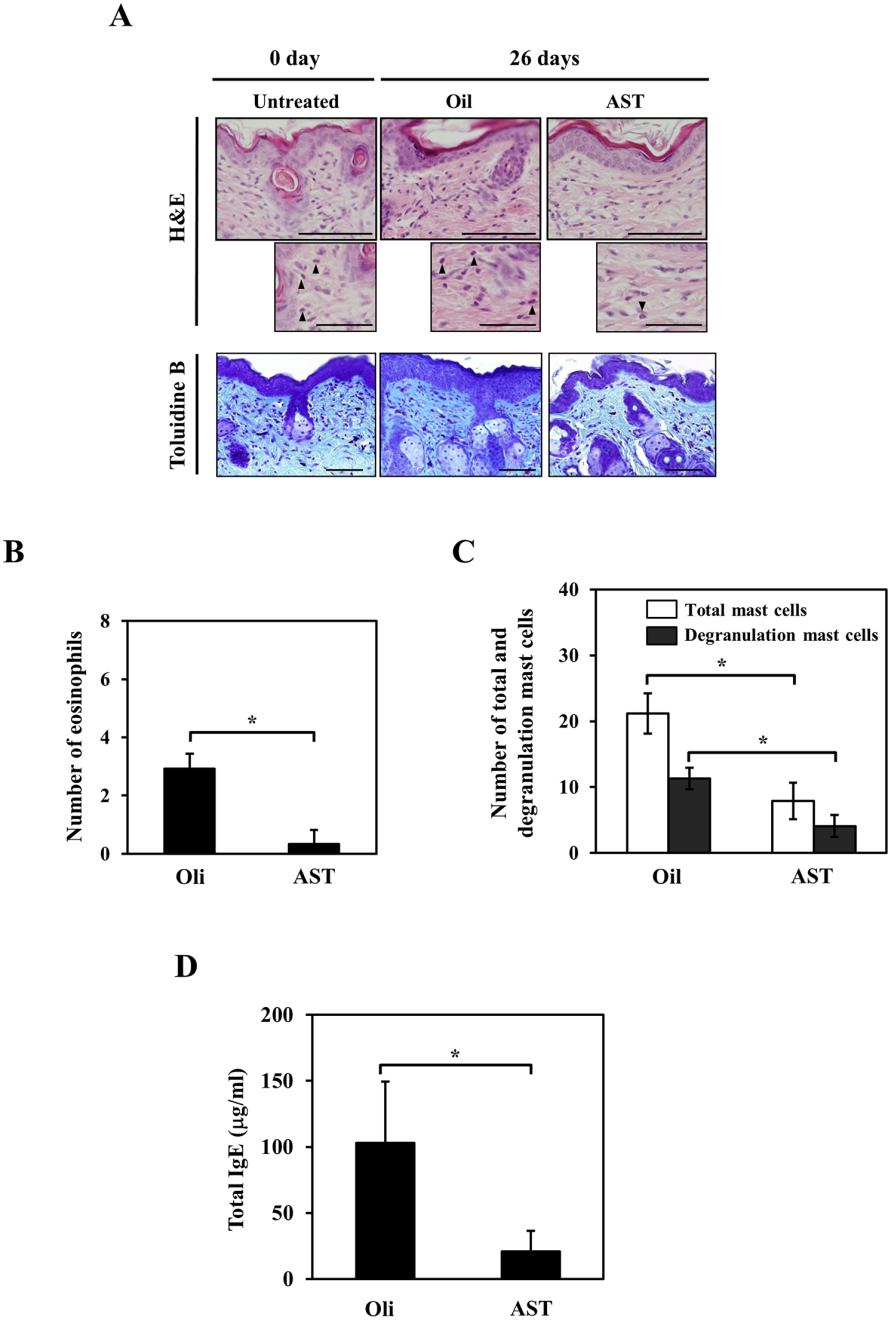
Effects of AST on the photomicrograph findings of the back skin and the serum total IgE levels in the NC/Nga mice. (A) The histological features of the NC/Nga mice orally administered AST or oil were determined using H&E and toluidine blue staining. Eosinophils are indicated by arrowheads. The experiments were repeated three times with similar results. Scale bar for large panels = 100 μm; scale bar for small panels = 10 μm. (B) The number of eosinophils in the skin lesions of NC/Nga mice orally administered AST were compared with those observed in the mice orally administered the oil vehicle. Each value represents the mean ± SD (n = 5; *p<0.05). (C) The number of total and degranulated mast cells in the skin lesions of the NC/Nga mice orally administered AST were compared with those observed in the mice orally administered oil. Each value represents the mean ± SD (n = 5; *p<0.05). (D) Relationship between AST treatment and the serum total IgE levels on day 26. The values represent the mean ± SD.

The infiltration of inflammatory cells, including mast cells and eosinophils, are histopathological changes that occur in the skin lesions of NC/Nga mice [14]. Skin specimens from the lesions of AST- or vehicle-treated mice were collected on day 0 and after the completion of the 26-day observation period. The number of eosinophils was significantly decreased in the dermis of the AST-treated mice compared to the vehicle-treated mice (Vehicle: 3.1 ± 0.6, AST: 0.6 ± 0.5, *p<0.05) (Fig 2A and 2B). Moreover, we observed a significant decrease in the number of total and de-granulated mast cells in the dermis of the AST-treated mice compared with the skin in the vehicle-treated mice (total, Vehicle: 21.2 ± 3.1, AST: 7.9 ± 2.7, de-granulated, Vehicle: 11.3 ± 1.6, AST: 4.1 ± 1.7, *p<0.001) (Fig 2A and 2C).

### AST treatment decreased the IgE level of NC/Nga mice

We investigated the effects of AST on the serum IgE levels of NC mice. The serum IgE levels were also significantly decreased in the mice treated with AST for 26 days compared to the vehicle-treated mice (*p<0.05) (Fig 2D).

### Effects of AST on the expression and production of pro-inflammatory cytokines and HDC in the skin of NC/Nga mice

**Fig 3:**
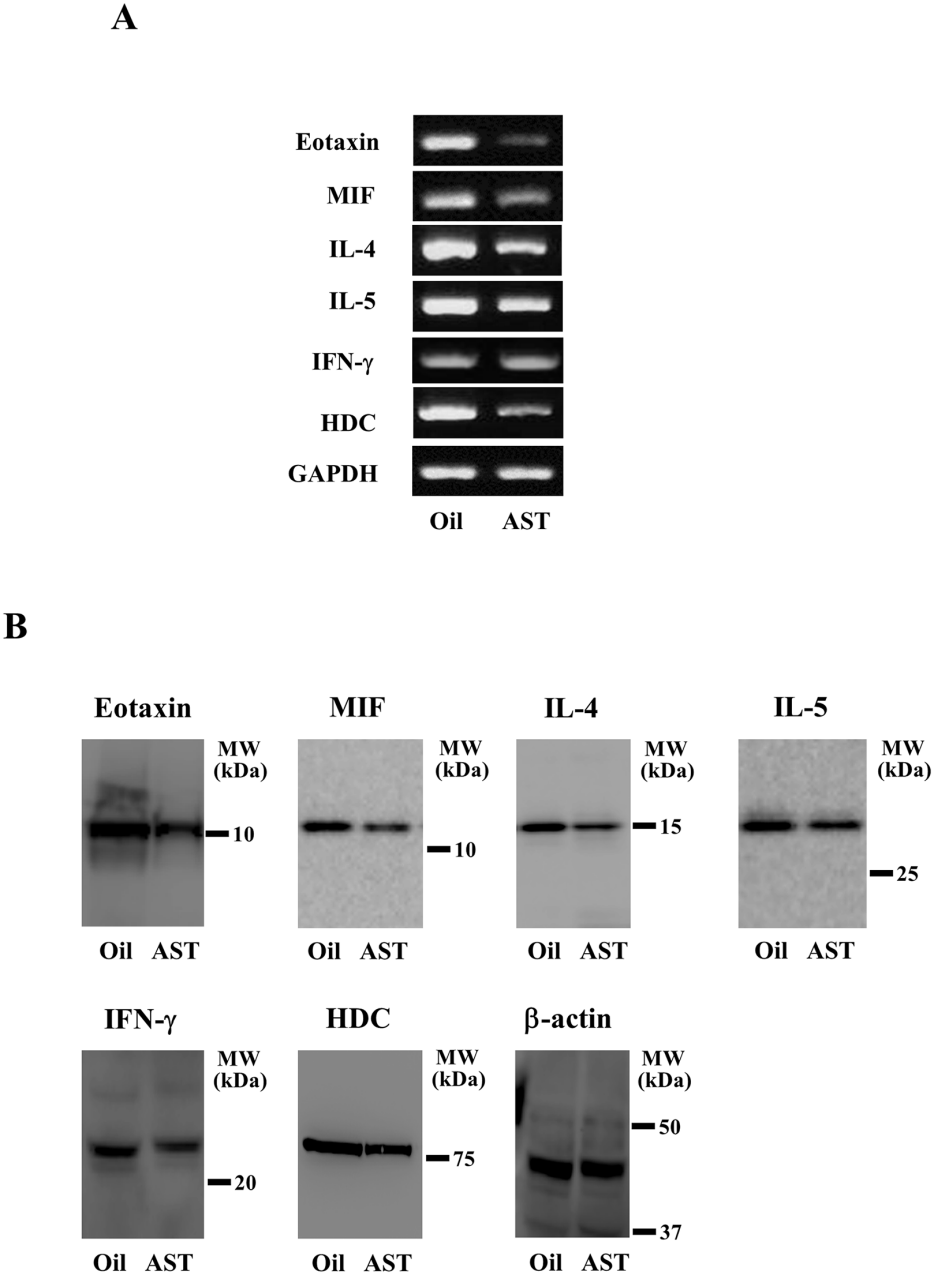
The effects of AST on the expression of pro-inflammatory mediators in the NC/Nga mouse skin. (A) The total RNA was isolated, and the mRNA levels of eotaxin, MIF, IL-4, IL-5, IFN-γ and HDC were detected by RT-PCR. Mouse GAPDH was used as an internal control for the RT-PCR. The data shown are representative of three independent experiments. (B) Skin lysates were prepared, and the protein levels were analyzed by a Western blot analysis using an anti-eotaxin, MIF, IL-4, IL-5, IFN-γ, HDC or anti-β-actin antibody. The anti-β-actin antibody was used as an internal control for the Western blot analysis. Cropped blots are shown, and all of the gels were run under the same conditions.

We then examined the effects of AST on the production of pro-inflammatory cytokines and the HDC expression in the mouse skin. AST treatment decreased the mRNA expression levels of eotaxin, MIF, IL-4, IL-5 and HDC (Fig 3A). In addition, a Western blot analysis revealed that AST treatment decreased the production of these cytokines and HDC in the mouse skin (Fig 3B). However, the expression of TNF-α and IL-1β remain unchanged following AST treatment (S2 Fig).

## Discussion

AD is a chronic or chronically relapsing inflammatory skin disease characterized by the presence of eczematous skin lesions with lichenification, pruritic excoriation, dry skin and susceptibility to skin infections [6]. Oxidative stress has been reported to play an important role in the pathophysiology and exacerbation of AD symptoms [2] and the use of antioxidants has been shown to be beneficial in protecting against the harmful effects of increased oxidative stress [16, 20].

AST is known to be a potent quencher of singlet oxygen and an efficient scavenger of superoxide anions. AST is expected to be a useful antioxidant for preventing oxidative stress, a causative factor in several diseases. Accordingly, in the present study, treatment with AST reduced the severity of skin irritation and improved the symptoms of dermatitis and pruritus in AD-model mice. In addition, AST has been reported to have beneficial effects (e.g. it inhibits the release of ROS) in the treatment of ischemic reperfusion injury [21] as well as neuroprotective effects in cases of Parkinson's disease [22] and preventive effects in cases of arteriosclerosis [23] and diabetic nephropathy [24]. Recently, our group demonstrated that the administration of AST induces a significant decrease in the protein content of inducible nitric oxide (iNOS) and cyclooxygenase (COX)-2 and inhibits the release of prostaglandin E2 from keratinocytes following ultraviolet irradation [16].

The skin inflammation observed in cases of AD is characterized histopathologically by the infiltration of T lymphocytes, monocytes, macrophages, eosinophils and mast cells. Increased numbers of eosinophils are commonly seen in many allergic diseases, including AD. These cells infiltrate the dermis in response to allergens and subsequently secrete eosinophil cationic protein (ECP) [25], which mediates the migration of other immune cells into developing skin lesions [26]. Similarly, mast cells, which are activated due to allergen-crosslinked IgE, enhance the development of Th2 cells and induce the release of chemokines, cytokines and granular mediators that participate in the development of AD-like skin lesions [27, 28]. In the present study, AST treatment effectively decreased the number of eosinophils and suppressed the AD allergic response.

IgE production plays an important role in the pathogenesis of skin diseases, as high serum IgE levels mediate the critical clinical characteristics of AD. Specifically, IgE binds with mast cells, thus causing the release of inflammatory mediators, which correlates with the severity of AD [29]. In patients with AD, there is an increased number of IgE-bearing Langerhans cells in the epidermis, which appear to play a crucial role in presenting cutaneous allergens to Th2 cells and may promote a clinical situation in which scratching is a known prerequisite for the development of AD skin lesions [30]. Therefore, IgE is considered to be one of the most significant therapeutic targets in AD. The present results demonstrated that AST treatment significantly improves the itching behaviour in mice, possibly due to its suppressive effects on the serum IgE level, eosinophils numbers and mast cells degranulation. Moreover, pro-inflammatory cytokines are believed to be important contributors to the pathogenesis of skin inflammation in patients with AD, a process that may depend on the duration of the skin lesion. The onset of AD involves a systemic Th2 response associated with eosinophilia and marked infiltration of Th2 cells into the skin lesions. These infiltrating T cells predominantly express IL-4, IL-5 and IL-13. Recently, the roles of cytokines in the induction of migration and accumulation of eosinophils into inflamed tissues have been reported. Important eosinophil chemoattractant cytokines include IL-5 and eotaxin [31]. Eotaxin is reportedly related to the development of eosinophilia in patients with allergic diseases, including AD and asthma [32, 33]. IL-5 also has important roles in eosinophil production and differentiation [34]. In contrast, recently it has been reported that Th2-mediated immune response is not necessary for the development of AD-like skin disease in NC mice [35], however other cytokines and mediators might also play their role. Therefore, the detailed pathophysiological mechanisms of AD are not completely clear, the activation of inflammatory cells and dysregulation of cytokine production appears to be common and may play a critical role in the pathogenesis of AD [21]. Therefore, the targeted inhibition of inflammatory cytokine production has long been regarded to be a possible therapeutic strategy for improving the clinical outcomes of AD. Consistent with the reports discussed above, AST treatment decreased the expression levels of eotaxin, MIF, HDC and Th2-type cytokines, including IL-4 and IL-5, in a murine model of AD and protected against the development of AD symptoms by inhibiting the production of inflammatory cytokines in this study. Recently, our group also reported similar findings showing that treatment with AST results in a reduction in the UV-induced protein and mRNA expression levels of MIF, IL-1βand TNF-αin HaCaT keratinocytes. It was suggested that protects agains UV-induced inflammation by decreasing inflammatory cytokines, and thereby inhibiting the apoptosis of keratinocytes [16].

In conclusion, we herein demonstrated that the oral administration of AST improves the symptoms of dermatitis and pruritus in cases of AD via the regulation of inflammatory effects and expression of inflammatory cytokines.

## Supporting Information

S1 Fig Photomicrographs of the mast cells. (A) A degranulated mast cell (arrow). (B) A non-degranulated mast cell (arrow). Toluidine blue staining, Scale bar = 50 μm.
(TIF)

S2 Fig The effects of AST on the expression of pro-inflammatory mediators in the NC/Nga mouse skin. Skin lysates were prepared, and the protein levels were analyzed by a Western blot analysis using an anti-TNF-α, IL-1β or anti-β-actin antibody. The anti-β-actin antibody was used as an internal control for the Western blot analysis. Cropped blots are shown, and all of the gels were run under the same conditions.
(TIF)
