# Supplementary figures and images for: Efficacy of Astaxanthin for the Treatment of Atopic Dermatitis in a Murine Model
Source: PLoS One. 2016 Mar 29;11(3):e0152288. doi: 10.1371/journal.pone.0152288 (PMC4811408; doi:10.1371/journal.pone.0152288)

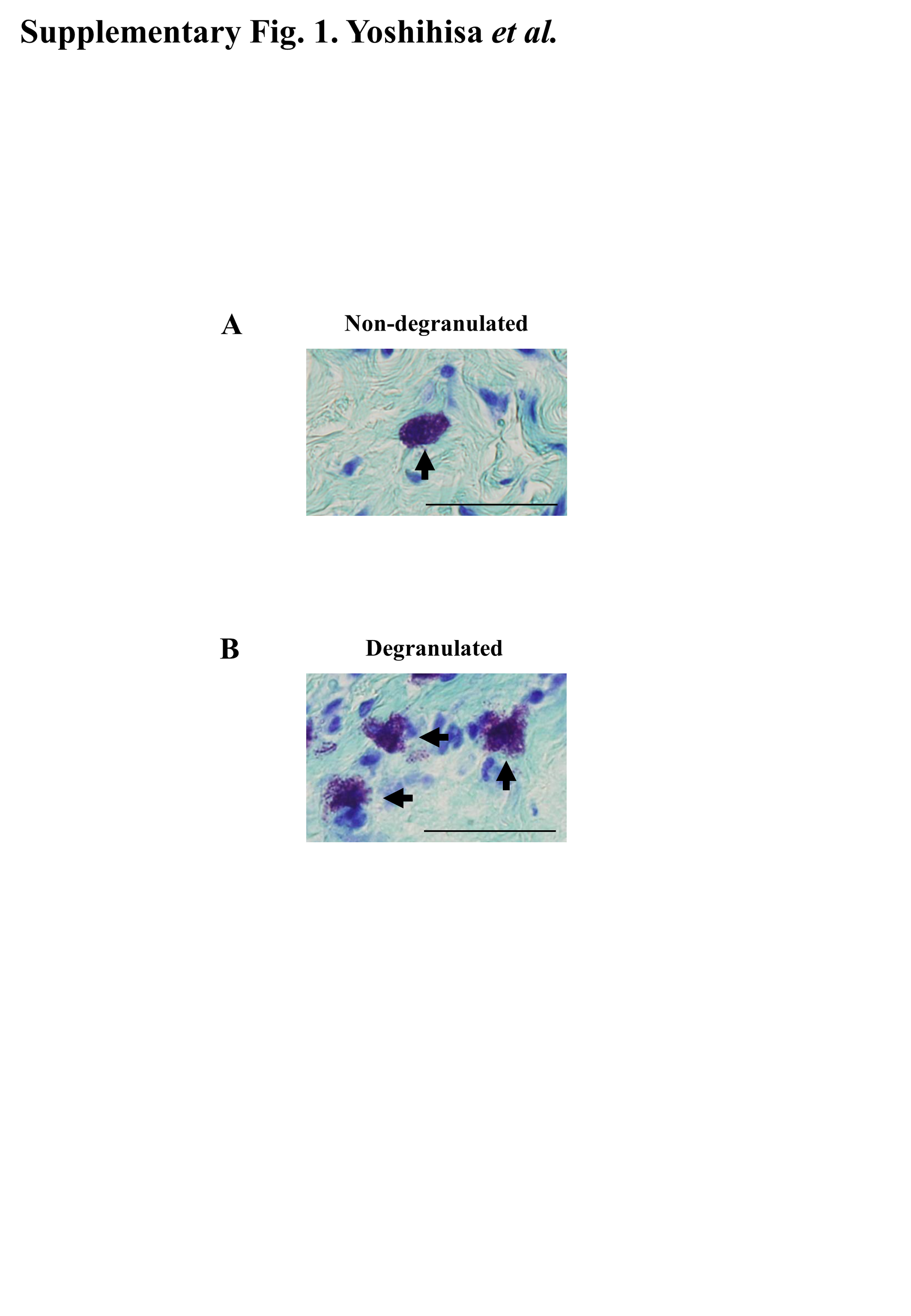

Supplement: S1 Fig — (A) A degranulated mast cell (arrow). (B) A non-degranulated mast cell (arrow). Toluidine blue staining, Scale bar = 50 μm. (TIF) [file pone.0152288.s001.tif]

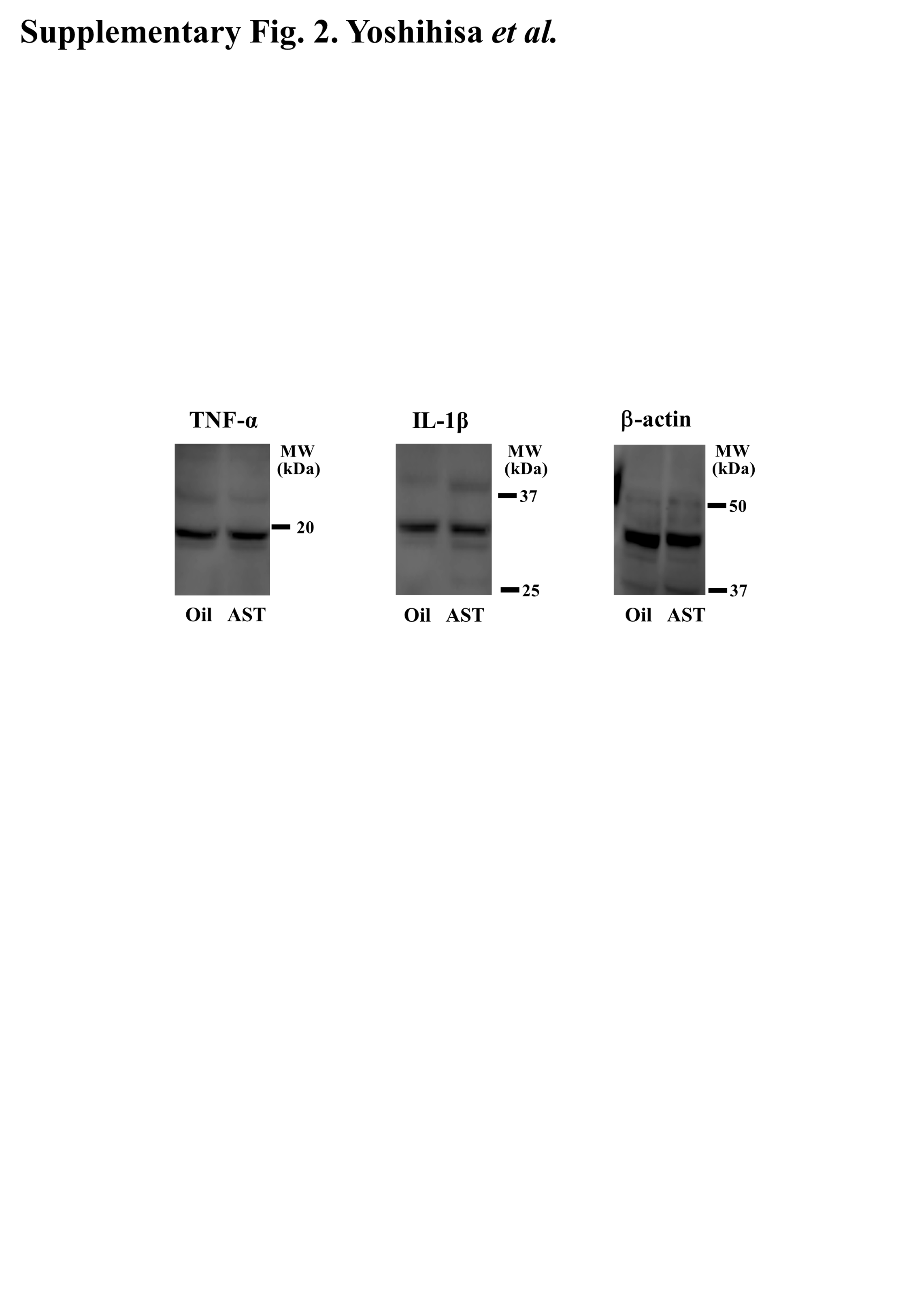

Supplement: S2 Fig — Skin lysates were prepared, and the protein levels were analyzed by a Western blot analysis using an anti-TNF-α, IL-1β or anti-β-actin antibody. The anti-β-actin antibody was used as an internal control for the Western blot analysis. Cropped blots are shown, and all of the gels were run under the same conditions. (TIF) [file pone.0152288.s002.tif]
